# Do the interactions between coital frequency, cervical length, and urogenital infection affect obstetric outcomes?

**DOI:** 10.4274/tjod.89106

**Published:** 2015-06-15

**Authors:** Yiğit Çakıroğlu, Şeyda Çalışkan, Emek Doğer, Şule Yıldırım Köpük, Devrim Dündar, Eray Çalışkan

**Affiliations:** 1 Kocaeli University Faculty of Medicine, Department of Obstetrics and Gynecology, Kocaeli, Turkey; 2 Sakarya University Faculty of Medicine Education and Research Hospital, Department of Clinical Microbiology, Sakarya, Turkey; 3 Kocaeli University Faculty of Medicine, Department of Clinical Microbiology, Kocaeli, Turkey

**Keywords:** preterm birth, urogenital infection, bacterial vaginosis, cervical length, coital frequency

## Abstract

**Objective:**

To determine whether interactions between coital frequency, cervical length, and urogenital infection affect obstetric outcomes.

**Materials and Methods:**

A total of 268 unselected pregnant women were recruited in the study. The study population consisted of four groups of women: group 1 (n=203) screened negative for bacterial vaginosis (BV) both in the first and second trimesters; group 2 (n=18) screened negative for BV in the first trimester but positive in the second trimester; group 3 (n=33) screened positive for BV in the first trimester but negative in the second trimester; and group 4 (n=14) screened positive for BV both in the first and second trimesters. Urine culture, cervico-vaginal cultures, and bacterial vaginosis were screened between 11-14 weeks and 20-24 weeks.

**Results:**

Two hundred fifty women were eligible for analysis in the study after lost-to-follow up patients were excluded. Previous abortion ≥1 and previous preterm delivery at 24-34 weeks ≥1 were statistically significantly higher in group 2. The number of patients who were diagnosed as having preterm premature rupture of membranes (PPROM) was statistically significantly higher in group 4. Sexual intercourse during the first trimester, cervical length during the second trimester, and history of preterm birth (PTB) were statistically significant risk factors for preterm birth <37 weeks (1.27; (1.12-1.44); 5.33; (1.84-15.41); 6.95; (1.58-30.54), respectively).

**Conclusion:**

Presence or treatment of BV did not influence rates of PTB. The probability of PPROM would be higher in patients who are BV positive both in the first and second trimesters.

## INTRODUCTION

Preterm birth (PTB) has been defined as birth before 37 weeks of gestation and is the leading cause of neonatal mortality and morbidity^(1)^. PTB can be divided into spontaneous or induced for maternal and fetal indications. There are multiple etiologic factors that may result in spontaneous preterm birth, including genital tract infections^(2)^. The mechanism responsible for PTB in pregnant women with genital tract infections might be ascending bacteria from the vagina to the chorioamniotic membranes and amniotic fluid^(3)^.

Among the microorganisms responsible for genital tract infections, bacterial vaginosis is the most commonly-associated microorganism-related clinical entity^(4)^. The prevalence of BV has been reported about to be 50% in pregnant women and 15-30% in non-pregnant women^(5,6)^. It is hard to investigate to the real prevalence and reported prevalence covers a wide range because most of cases of BV are asymptomatic and the populations that have been investigated were highly selected groups.

The effect of coitus on the outcome of pregnancies remains inconclusive. Some studies have reported no relationship with coital activity and PTB^(7,8)^. Read et al. reported no association between frequent sexual intercourse and PTB, but contrary to previous authors, they reported an increased risk of PTB in the presence of microorganisms^(9)^.

Either prostaglandin in semen might have a direct effect on the cervix, or coital activity might influence bacterial colonization and result in PTB^(10)^.

Cervical length assessment via ultrasound can be performed for the prediction of PTB in the first and/or second trimesters^(11)^. Evaluation of cervical length in patients with a history of PTB or in asymptomatic patients might have small differences^(12)^. It is not yet clear whether the presence or absence of a vaginal microorganism might have effects on cervical shortening.

In our study, we aimed to determine whether interactions between coital frequency, cervical length, and urogenital infection affected obstetric outcomes.

## MATERIALS AND METHODS

A total of 268 unselected pregnant women who were admitted to the obstetrics unit of a tertiary center at at Kocaeli University between July 2007 and September 2011 were invited to participate in the study. The general PTB rate was 12.7%^(13)^. The sample size needed to recruit was calculated as t2*pq/d2 with a 95% probability to predict this prevalance (1.962*0.1*0.9/0.052=140). The minimum number of patients to be recruited for the study was 140 according to the formula and we recruited 250 patients. Two hundred fifty women were eligible for analysis in the study after the lost-to-follow-up patients were excluded. The local ethics committee approved the study. Written informed consent was obtained from all the patients who participated in the study.

In this study, the primary outcome measure was to determine whether interactions between coital frequency, cervical length, and urogenital infection affected obstetric outcomes.

Criteria for enrollment included all the healthy pregnant women who were admitted during the first trimester. Exclusion criteria included: 1) multifetal pregnancy, 2) intrauterine fetal death, 3) fetal abnormality, and 4) systemic maternal disease. Women with a prior PTB were not excluded from the study.

After patients were evaluated according to the inclusion and exclusion criteria, a total of 268 patients were investigated. The study population consisted of four groups of women: women who screened negative for bacterial vaginosis both in the first and second trimesters; (group 1; n=203), those who screened negative for bacterial vaginosis in the first trimester but positive in the second trimester (group 2; n=18), those who screened positive for bacterial vaginosis in the first trimester but negative in the second trimester (group 3; n=33); and those who screened positive for bacterial vaginosis both in the first and second trimesters (group 4; n=14). Data analysis was performed after excluding patients who were lost-to-follow-up (6 patients in group 1, five patients in group 2, four patients in group 3, and three patients in group 4).

All patients underwent gynecologic examination with a sterile speculum and were investigated for vaginal discharge. Urine culture, cervico-vaginal cultures, and bacterial vaginosis were screened between 11-14 weeks and 20-24 weeks. A polyester swab taken from the junction of the upper third and lower two thirds of the lateral vaginal wall was rolled on a glass slide. The slides were Gram stained and interpreted in accordance with the criteria of Nugent et al.^(14)^. Bacterial vaginosis was diagnosed if the Gram stain score was 7 to 10. Control vaginal samples were obtained one week after completion of the therapy. Patients with bacterial vaginosis were treated with ornidazole 500 mg for 5 days (Ornisid, Abdi İbrahim, Turkey). Vaginal swabs were directly inoculated onto Sabouraud dextrose agar (SDA) plates (Oxoid), which were incubated at 37 °C for 48 h to isolate *Candida spp*. All strains of *Candida spp*. were identified based on the germ tube test, and morphologic characteristics on Cornmeal Tween-80 agar and API 20C AUX system (BioMerieux).

Ultrasonographic examinations were performed with (Medison Sonoace 8x Ultrasound Machine, Inc., 4-8 MHz) a transvaginal probe. A single specialist (EC) performed the measurements in order to eliminate the possibility for interobserver variability in measurement technique. The specialist was blinded to the women’s previous cervical length records. After the patient emptied her bladder, she was placed in the lithotomy position. Transvaginal ultrasonographic measurements of the cervix were made with a standard technique, as previously described by Iams et al.^(15)^. The internal cervical os was identified using a sagittal plane view, and the calipers were placed at the furthest points between the internal and external cervical os. When funneling was present, we measured the distance over which the endocervical walls were juxtaposed. Three measurements were recorded for each and the shortest measurements were used. According to the trial protocol, cervical length was measured first at 11-14 weeks corresponding to routine first trimester nuchal translucency screening, the second was at 18-20 weeks corresponding to the triple test and abnormality screening, and lastly at 28-32 weeks of gestation.

The statistical analysis of the data was performed using the Statistical Package for Social Sciences for Windows (SPSS, Chicago, IL, USA). Results were reported as mean ± standard deviation and percentages. Chi-square test was used to compare categorical variables. Student’s T-test was used for comparing continuous variables. Binary logistic regression analysis first with Enter and then with forward Wald methods were used to determine risk factors of PTB (<37 weeks of gestation) as a dicotomous outcome variable. The equation was calculated by independent continous variables such as mean coital frequency/week between admission to 14 weeks, mean coital frequency/week between 14 weeks to 28 weeks and categorical variables such as cervical length ≥30 mm or <30 mm at 11-14 weeks, cervical length ≥30 mm or <30 mm at 18-20 weeks, presence or absence of an urinary infection in the first and second trimesters, vaginal symptoms in the first and second trimesters, candidial infection in the first and second trimesters, bacterial vaginosis in the first and second trimesters and history of presence of PTB. Statistical significance was considered p values less than 0.05.

## RESULTS

From a total of 268 women, after lost-to-follow-up patients were excluded, 250 were eligible for analysis in the study. Selected variables according to the groups are presented in Table 1. Previous abortion ≥1 and previous preterm delivery at 24-34 weeks ≥1 were statistically significantly higher in group 2 when compared with other groups. Coital frequency, cervical length measurements and urinary infection positivity were similar in between the groups. While candida positivity was similar between the groups at the first and second trimesters, it was statistically significantly higher in groups 2 and 4 when compared with other groups.

Maternal and fetal variables according to the groups are shown in Table 2. The number of patients who were diagnosed as having preterm premature rupture of membranes (PPROM) was statistically significantly higher in group 4 when compared with other groups. When birth weight of newborns was compared between the groups, it was statistically significantly higher in groups 1 and 2. Neonatal intensive care unit (NICU) admission was also compared between the groups and the analysis revealed statistically significantly higher number of admissions in groups 3 and 4.

The risk factors for PTB <37 weeks of gestation are demonstrated on Table 3. Sexual intercourse during the first trimester, cervical length during the second trimester and history of PTB were statistically significant risk factors for PTB <37 weeks.

## DISCUSSION

In the present study, we assessed the presence of bacterial vaginosis in the first and second trimesters of pregnancy and evaluated the effects and associations of BV, cervical length, and coital frequency on PTB.

A strong positive correlation was defined between BV and PTB^(16)^. The incidence of PTB among patients with BV was about 1.4-6.9%^(17)^. Independent from the patients’ symptoms, no women with BV gave birth prematurely. Ascending microorganisms may lead to chorioamnionitis, or mucosal permeability may alter due to BV, or local immunity may overrespond, and all of these mechanisms may result in PTB. Also, the earlier the bacterial colonization builds, the stronger the risk of PTB^(18)^. Thus, the frequency of PTB may differ according to the time interval that the vagina, cervix or the fetal parts are exposed to microorganisms. In our study, PPROM was highest in patients who were BV positive both in the first and second trimesters, followed by patients who were BV positive in the first trimester but negative in the second trimester (45.4% and 13.7%, respectively).

In the present study, patients with BV were treated during pregnancy. Berghella et al. investigated the association of PTB and sexual intercourse in patients treated for BV^(19)^. Interestingly, their results indicated a decreased incidence of PTB in patients who had intercourse between the second and third trimesters. The authors commented that this was a result of more liberal sexual intercourse in patients without a history of PTB. Nygren et al. and James et al. reported no effects of routine screening and treatment of BV in asymptommatic pregnant women^(4,16)^. Our results showed that even though the incidence of PTB was highest in patients who were BV positive in the first and second trimesters, it did not reach statistical significance. In our multivariate analysis, bacterial vaginosis was not found to be a significant risk factor for PTB, which might be due to the effective treatment of BV. Laxmi et al. researched adverse fetomaternal outcomes in patients with bacterial vaginosis and reported BV as a risk factor for adverse outcomes^(5)^. Our results indicated statistically significantly increased neonatal intensive care unit admission in patients who were BV positive in the first trimester but negative in the second trimester, followed by patients with BV positive both in the first and second trimesters (16.7% and 11.1%, respectively). Neonatal sepsis and respiratory distress syndrome were not detected in any of the newborns in any groups.

Cervical length assessment has been investigated in the literature and has been used as a useful adjunct for the prediction of PTB^(20,21,22)^. Mancosu et al. evaluated whether cervical length would shorten more rapidly in patients with BV^(23)^. After adjustment of variables, they reported no effect of BV on cervical shortening. In our study, cervical length measurements in all the trimesters in all the groups were not statistically significantly different. Logistic regression analysis revealed a statistically significant effect of cervical length measurement on PTB.

Effect of coitus on PTB has been investigated in the literature. Kurki et al. investigated the association of coitus, bacterial vaginosis, and PTB^(7)^. They concluded that coitus during pregnancy increased neither bacterial vaginosis nor PTB. Similarly, Yost et al. researched the effect of coitus in early pregnancy on PTB and reported no association between the two parameters^(8)^. Read et al. reported no association between frequent sexual intercourse and PTB, but contrary to previous authors, they reported an increased risk of PTB in the presence of microorganisms^(9)^. In our study, coital frequency had no influence on BV in all groups in all trimesters. Also, only coital frequency in the first trimester was associated with an increased risk of PTB in the regression analysis model, but coital frequency in the second trimester was not associated with an increased risk of PTB. Our results indicated no effect of coitus on PTB after the first trimester.

In conclusion, our results revealed that presence of or treatment of BV did not influence rates of PTB. The probability of PPROM would be higher in patients with BV positive both in the first and second trimesters. Coital frequency in the first trimester, cervical length in the second trimester, and history of PTB are variables that may affect rates of PTB.

## Figures and Tables

**Table 1:**
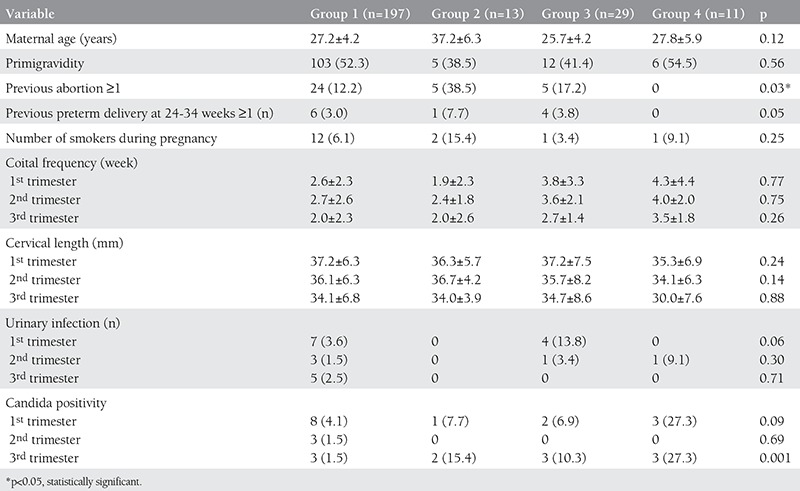
Maternal variables according to the groups (values are n, mean (± standard deviation) or n/N (%)).

**Table 2:**
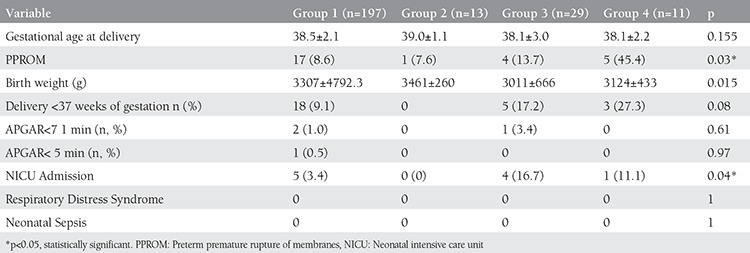
Maternal and fetal variables according to data of women who were screened negative for bacterial vaginosis both in the first and second trimesters shortened as (-/-) for group 1, group 2 (-/+), group 3 (+/-) and group 4 (+/+) (values are n, mean (± standard deviation) or n/N (%)).

**Table 3:**
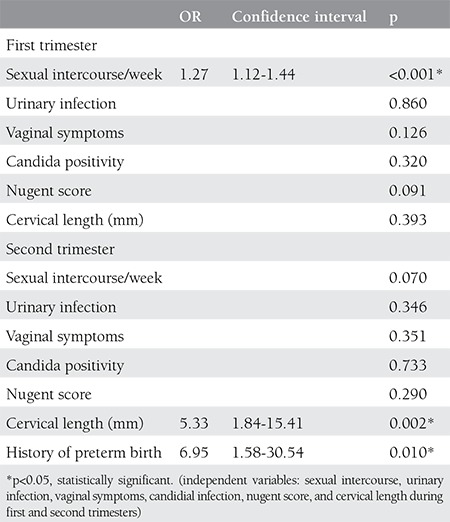
The linear regression model for predictors of Preterm birth <37 weeks of gestation
